# Supplementary material for: NRF2 polymorphism and susceptibility to ischemic stroke in a Chinese population
Source: PeerJ. 2025 Jul 24;13:e19742. doi: 10.7717/peerj.19742 (PMC12296571; doi:10.7717/peerj.19742)
Supplement: Supplemental Information 3 — *, Adjusted by age, gender and diabetes mellitus; CI, Confidence Interval; HWE, Hardy–Weinberg equilibrium; IS, ischemic stroke; OR, Odds Ratio; SNP, Single-Nucleotide Polymorphism; Bold: p < 0.05 [file peerj-13-19742-s003.docx]

**Table S1.** Data on *NRF2* SNPs in control participants and patients with ischemic stroke.

| **SNPs** | **Model** | **Genotypes** | **Control group** | **IS group** | **OR* (95% CI)** | ***p**** |
| --- | --- | --- | --- | --- | --- | --- |
| rs13005431 | Co-dominant | CC | 58 | 66 | Ref |  |
|  |  | CT | 59 | 76 | 1.157 (0.701–1.910) | 0.568 |
|  |  | TT | 24 | 17 | 0.656 (0.317–1.358) | 0.256 |
|  | Dominant | CC | 58 | 66 | Ref |  |
|  |  | CT+TT | 83 | 93 | 1.0113 (0.632–1.622) | 0.958 |
|  | Recessive | CT+CC | 117 | 142 | Ref |  |
|  |  | TT | 24 | 17 | 0.608 (0.308–1.199) | 0.151 |
|  | Overdominant | TT+CC | 82 | 83 | Ref |  |
|  |  | CT | 59 | 76 | 1.286 (0.806–2.051) | 0.292 |
|  | Allele | C | 175 | 208 | Ref |  |
|  |  | T | 107 | 110 | 0.889 (0.635–1.244) | 0.493 |
|  | HWE *P* |  | 0.648 | 0.880 |  |  |
| rs4893819 | Co-dominant | TT | 59 | 62 | Ref |  |
|  |  | CT | 60 | 78 | 1.253 (0.758–2.071) | 0.379 |
|  |  | CC | 22 | 19 | 0.831 (0.402–1.717) | 0.617 |
|  | Dominant | TT | 59 | 62 | Ref |  |
|  |  | CT+CC | 82 | 97 | 1.138 (0.709–1.827) | 0.591 |
|  | Recessive | CT+TT | 119 | 140 | Ref |  |
|  |  | CC | 22 | 19 | 0.738 (0.376–1.450) | 0.378 |
|  | Overdominant | TT+CC | 81 | 81 | Ref |  |
|  |  | CT | 60 | 78 | 1.313 (0.822–2.096) | 0.254 |
|  | Allele | T | 178 | 202 | Ref |  |
|  |  | C | 104 | 116 | 0.990 (0.706–1.388) | 0.953 |
|  | HWE *P* |  | 0.772 | 0.871 |  |  |
| rs6721961 | Co-dominant | GG | 80 | 90 | Ref |  |
|  |  | GT | 51 | 62 | 1.073 (0.654–1.762) | 0.780 |
|  |  | TT | 10 | 7 | 0.587 (0.206–1.676) | 0.320 |
|  | Dominant | GG | 80 | 90 | Ref |  |
|  |  | GT+TT | 61 | 69 | 0.994 (0.617–1.600) | 0.979 |
|  | Recessive | GT+GG | 131 | 152 | Ref |  |
|  |  | TT | 10 | 7 | 0.570 (0.204–1.593) | 0.284 |
|  | Overdominant | GG+TT | 90 | 97 | Ref |  |
|  |  | GT | 51 | 62 | 1.130 (0.695–1.836) | 0.623 |
|  | Allele | G | 211 | 242 | Ref |  |
|  |  | T | 71 | 76 | 0.916 (0.619–1.355) | 0.659 |
|  | HWE *P* |  | 0.946 | 0.803 |  |  |
| rs35652124 | Co-dominant | TT | 36 | 28 | Ref |  |
|  |  | TC | 54 | 78 | 1.869 (1.007–3.469) | **0.048** |
|  |  | CC | 51 | 53 | 1.335 (0.704–2.532) | 0.376 |
|  | Dominant | TT | 36 | 28 | Ref |  |
|  |  | TC+CC | 105 | 131 | 1.607 (0.909–2.841) | 0.103 |
|  | Recessive | TC+TT | 90 | 106 | Ref |  |
|  |  | CC | 51 | 53 | 0.879 (0.540–1.432) | 0.605 |
|  | Overdominant | TT+CC | 87 | 81 | Ref |  |
|  |  | TC | 54 | 78 | 1.562 (0.974–2.504) | 0.064 |
|  | Allele | T | 126 | 134 | Ref |  |
|  |  | C | 156 | 184 | 0.911 (0.669–1.248) | 0.563 |
|  | HWE *P* |  | 0.165 | 0.999 |  |  |
| rs6726395 | Co-dominant | GG | 58 | 67 | Ref |  |
|  |  | AG | 59 | 76 | 1.144 (0.694–1.887) | 0.597 |
|  |  | AA | 24 | 16 | 0.620 (0.297–1.294) | 0.203 |
|  | Dominant | GG | 58 | 67 | Ref |  |
|  |  | AG+AA | 83 | 92 | 0.994 (0.621–1.591) | 0.979 |
|  | Recessive | AG+GG | 117 | 143 | Ref |  |
|  |  | AA | 24 | 16 | 0.578 (0.291–1.151) | 0.119 |
|  | Overdominant | GG+AA | 82 | 83 | Ref |  |
|  |  | AG | 59 | 76 | 1.286 (0.806–2.051) | 0.292 |
|  | Allele | G | 175 | 210 | Ref |  |
|  |  | A | 107 | 108 | 0.871 (0.621–1.221) | 0.423 |
|  | HWE *P* |  | 0.648 | 0.840 |  |  |
| rs2364723 | Co-dominant | CC | 34 | 41 | Ref |  |
|  |  | CG | 70 | 87 | 1.096 (0.622–1.931) | 0.752 |
|  |  | GG | 37 | 31 | 0.723 (0.368–1.422) | 0.348 |
|  | Dominant | CC | 34 | 41 | Ref |  |
|  |  | CG+GG | 107 | 118 | 0.967 (0.564–1.655) | 0.901 |
|  | Recessive | CG+CC | 104 | 18 | Ref |  |
|  |  | GG | 37 | 31 | 0.680 (0.390–1.185) | 0.174 |
|  | Overdominant | CC+GG | 71 | 72 | Ref |  |
|  |  | CG | 70 | 87 | 1.279 (0.803–2.037) | 0.300 |
|  | Allele | C | 138 | 169 | Ref |  |
|  |  | G | 144 | 149 | 0.855 (0.610–1.199) | 0.364 |
|  | HWE *P* |  | 0.998 | 0.678 |  |  |
| rs2706110 | Co-dominant | CC | 87 | 96 | Ref |  |
|  |  | CT | 48 | 59 | 1.077 (0.660–1.757) | 0.767 |
|  |  | TT | 6 | 4 | 0.568 (0.150–2.145) | 0.404 |
|  | Dominant | CC | 87 | 96 | Ref |  |
|  |  | CT+TT | 54 | 63 | 1.020 (0.634–1.642) | 0.934 |
|  | Recessive | CT+CC | 135 | 155 | Ref |  |
|  |  | TT | 6 | 4 | 0.552 (0.148–2.060) | 0.377 |
|  | Overdominant | CC+TT | 93 | 100 | Ref |  |
|  |  | CT | 48 | 59 | 1.109 (0.683–1.801) | 0.675 |
|  | Allele | C | 222 | 251 | Ref |  |
|  |  | T | 60 | 67 | 0.955 (0.630–1.449) | 0.829 |
|  | HWE *P* |  | 0.991 | 0.541 |  |  |

*, Adjusted by age, gender and diabetes mellitus.

CI, Confidence Interval; HWE, Hardy–Weinberg equilibrium; IS, ischemic stroke; OR, Odds Ratio; SNP, Single-Nucleotide Polymorphism. Bold: p<0.05.
